# miR-378a-3p modulates tamoxifen sensitivity in breast cancer MCF-7 cells through targeting *GOLT1A*

**DOI:** 10.1038/srep13170

**Published:** 2015-08-10

**Authors:** Kazuhiro Ikeda, Kuniko Horie-Inoue, Toshihide Ueno, Takashi Suzuki, Wataru Sato, Takashi Shigekawa, Akihiko Osaki, Toshiaki Saeki, Eugene Berezikov, Hiroyuki Mano, Satoshi Inoue

**Affiliations:** 1 Division of Gene Regulation and Signal Transduction, Research Center for Genomic Medicine, Saitama Medical University, Saitama, Japan; 2 Department of Cellular Signaling, Graduate School of Medicine, The University of Tokyo, Tokyo, Japan; 3 Departments of Pathology and Histotechnology, Tohoku University, Graduate School of Medicine, Miyagi, Japan; 4 Department of Breast Oncology, International Medical Center, Saitama Medical University, Saitama, Japan; 5 European Research Institute for the Biology of Ageing, University of Groningen, University Medical Center Groningen, Groningen, The Netherlands; 6 Departments of Anti-Aging Medicine and Geriatric Medicine, Graduate School of Medicine, The University of Tokyo, Tokyo, Japan

## Abstract

Breast cancer is a hormone-dependent cancer and usually treated with endocrine therapy using aromatase inhibitors or anti-estrogens such as tamoxifen. A majority of breast cancer, however, will often fail to respond to endocrine therapy. In the present study, we explored miRNAs associated with endocrine therapy resistance in breast cancer. High-throughput miRNA sequencing was performed using RNAs prepared from breast cancer MCF-7 cells and their derivative clones as endocrine therapy resistant cell models, including tamoxifen-resistant (TamR) and long-term estrogen-deprived (LTED) MCF-7 cells. Notably, miR-21 was the most abundantly expressed miRNA in MCF-7 cells and overexpressed in TamR and LTED cells. We found that miR-378a-3p expression was downregulated in TamR and LTED cells as well as in clinical breast cancer tissues. Additionally, lower expression levels of miR-378a-3p were associated with poor prognosis for tamoxifen-treated patients with breast cancer. *GOLT1A* was selected as one of the miR-378a-3p candidate target genes by *in silico* analysis. *GOLT1A* was overexpressed in breast cancer specimens and *GOLT1A*-specific siRNAs inhibited the growth of TamR cells. Low *GOLT1A* levels were correlated with better survival in patients with breast cancer. These results suggest that miR-378a-3p-dependent *GOLT1A* expression contributes to the mechanisms underlying breast cancer endocrine resistance.

Estrogen is an important endocrine hormone that regulates the growth and differentiation of the normal mammary gland^1^,^2^. The hormone also plays a critical role in the development and progression of breast cancer, which is one of the most common cancers among women^1^,^2^. The estrogen receptor α (ERα) is a member of the nuclear receptor superfamily that functions as transcription factors^3^,^4^,^5^. ERα mediates various functions of estrogen in its normal and malignant target tissues, including breast cancer^3^,^4^,^5^. Determination of ERα status is clinically used as a prognostic and predictive factor in the management of breast cancer. Approximately 85% of breast cancers are ERα positive and can be treated with endocrine therapy using anti-estrogens such as tamoxifen or aromatase inhibitors^6^,^7^,^8^. Tamoxifen has been used for years for adjuvant treatment, which significantly reduces the risk of recurrence of breast cancer^9^. Despite the obvious benefits of tamoxifen, approximately 40% of patients with early-stage breast cancer treated with tamoxifen as adjuvant therapy would eventually suffer from the relapse with tamoxifen-resistant disease^10^. Thus, studies have been performed to elucidate the molecular mechanisms underlying endocrine resistance; however, only several potential targets and signaling pathways have been revealed^11^,^12^,^13^. Therfore, a better understanding of the tamoxifen-resistant mechanism may provide novel strategies to overcome tamoxifen resistance in breast cancer.

MicroRNAs (miRNAs) are small noncoding RNAs consisting of an average of 22 nucleotides. miRNAs can function as post-transcriptional regulators by binding to 3′-untranslated regions (3′-UTRs) of their target mRNAs in sequences that have imperfect or perfect complementarity, repressing the translation or degradation of their target mRNAs^14^,^15^. Nowadays, particular attention has been paid to the deregulation of miRNAs in tumor progression and metastasis as one of the new transcriptional regulators involved in cancer biology^16^,^17^,^18^. While the molecular mechanisms underlying tamoxifen resistance in terms of its key regulators and signaling events remain to be elucidated, miRNAs could be novel therapeutic targets for endocrine therapy resistant cancers.

Several studies have recently reported the role of miRNAs in tamoxifen resistance. These studies provide a list of miRNAs potentially involved in tamoxifen resistance, including miR-873^19^, miRNAs-221/222^20^,^21^,^22^, miR-519a^23^, miR-126 and miR-10a^24^, miRNA-200b and miR-200c^25^, miR-146a, -27a, -145, -21, -155, -15a, -125b, and let-7s^26^,^27^, miR-375^28^, miR-451^29^, miR-342^30^, and miR-574-3p^31^. For example, miR-873 is downregulated in tamoxifen-resistant MCF-7 cells and in breast cancer tissues compared with normal tissue. miR-873 decreases ER transcriptional activity through the modulation of ERα phosphorylation and inhibits the proliferation of breast cancer cells via targeting cyclin-dependent kinase 3 (CDK3)^19^. Loss of miR-375 expression is reported in tamoxifen-resistant breast cancer cells derived from long-term passage of MCF-7 cells with tamoxifen^28^. Re-expression of miR-375 sensitized tamoxifen-resistant breast cancer cells to tamoxifen and partly reversed the epithelial-mesenchymal transition (EMT)-like properties. This report showed that *MTDH*, which encodes metadherin, is a direct target of miR-375^28^. miR-574-3p has been identified as a tamoxifen response-related miRNAs in breast cancer cells by miRNA library-based functional screening^31^. miR-574-3p potentially targets clathrin heavy chain (CLTC) whose expression is associated with tamoxifen sensitivity in tamoxifen-resistant breast cancer cells.

In the present study, we performed high-throughput sequencing of miRNAs in human breast cancer MCF-7 cells and its derivative tamoxifen-resistant TamR cells and long-term estrogen-deprived (LTED) cells. Differentially expressed miRNAs were identified by comparing miRNA expression profiles among those cells. miR-21 was determined as an upregulated miRNA in TamR and LTED cells, and let-7s (let-7a and f) and miR-378a-3p were identified as downregulated ones. We further studied the expression of miR-378a-3p in clinical breast cancer specimens and found that the miRNA was downregulated in cancer tissues compared to adjacent normal tissues. Additionally, lower miR-378a-3p expression levels were associated with poor prognosis for tamoxifen-treated patients with breast cancer. By *in silico* analysis and luciferase reporter assay for miRNA binding sites, golgi transport 1A (*GOLT1A*) was identified as a candidate miR-378a-3p target. Loss-of-function experiments for GOLT1A in TamR cells resulted in a decrease of tamoxifen-resistant cell growth. Low *GOLT1A* levels were also shown to be correlated with better survival in patients with breast cancer. These results show that miR-378a-3p and its target *GOLT1A* can provide new insights into the signaling pathways associated with tamoxifen resistance in breast cancer and could be applied to the development of alternative diagnostic and therapeutic options for advanced breast cancer.

## Results

### Identification of miRNAs differentially expressed in breast cancer cells and endocrine therapy-resistant cells using high throughput sequencing

**Figure 1:**
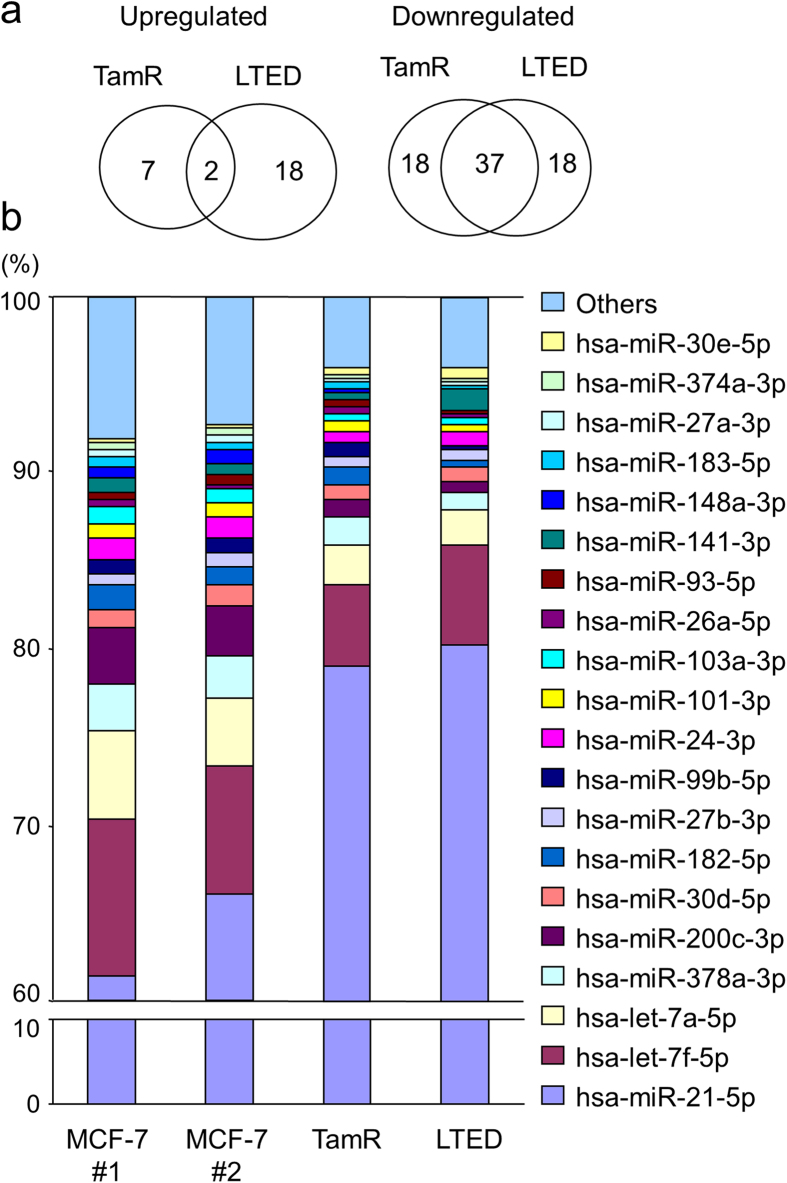
miRNA expression profiles of breast cancer MCF-7 and its derivative endocrine therapy resistant cells. (**a**) Differentially expressed miRNAs in tamoxifen-resistant breast cancer cells (TamR) and long-term estrogen-deprived breast cancer cells (LTED) compared to their parental MCF-7 cells. TamR and LTED were established from MCF-7 cells (see Materials and Methods). Small RNA libraries were constructed from MCF-7, TamR, and LTED cells and sequenced using the Illumina GAIIx. (**b**) Percentages of top 20 miRNAs abundantly expressed in MCF-7 and endocrine therapy resistant cells. Percentages in distinctive cell lines were determined by a read count for each indicated miRNA normalized to the total count of 94 selected miRNAs (>10 reads) listed in Supplementary Table S1.

To identify novel miRNAs associated with endocrine therapy-resistance of breast cancer cells, tamoxifen-resistant (TamR) cells^32^ were obtained from MCF-7 cells by long-term (>3 months) culture with 1 μM tamoxifen and long-term estrogen-deprived (LTED) cells^33^ were established from MCF-7 cells by culturing them in phenol red-free Dulbecco’s Modified Eagle Medium (DMEM) supplemented with 10% dextran-charcoal stripped fetal bovine serum (dcc FBS). High-throughput miRNA sequencing was performed using RNAs prepared from parental MCF-7, TamR and LTED cells. Mapping of small RNA reads on the human genome (NCBI35 assembly) and prediction of novel miRNAs were performed as described previously^34^. An average of 4.6 million reads per sample was mapped on miRNAs. To compare miRNA expression profiles of these cells, a read count for each miRNA was shown as a frequency against the total mapped read count per sample. miRNAs exhibiting >10 reads were selected and resulted in a list of 94 annotated miRNAs in MCF-7, TamR, and LTED cells (Supplementary Table 1). No confident novel miRNA was predicted from the data. Among these annotated miRNAs, 9 and 20 miRNAs were upregulated (>1.2-fold) in TamR and LTED cells, respectively, compared with parental MCF-7 cells. Fifty five miRNAs were downregulated (<0.7-fold) in both TamR and LTED cells compared with their parental cells. Two and 37 miRNAs were commonly upregulated and downregulated, respectively, in TamR and LTED cells compared with their parental cells (Fig. 1a). We found that the percentages of miR-21 counts were approximately two-third of the total counts for 94 miRNAs in MCF-7 cells and >75% of the total counts in TamR and LTED cells (Fig. 1b). miR-21 is a known onco-miRNA in cancer, and its overexpression is shown to be associated with advanced clinical stage, lymph node metastasis, and poor prognosis in cancer patients, including breast cancer patients^35^,^36^,^37^,^38^,^39^. It has been also shown that miR-21 regulates the expression of target genes, including phosphatase and tensin homolog (PTEN), programmed cell death 4 (PDCD4), and tropomyosin 1 (TPM1) in breast cancer cells^40^,^41^,^42^.

Let-7f and let-7a, members of let-7 family, were also identified as abundantly and differentially expressed miRNAs among these cells. The expression levels of both let-7f and let-7a were decreased in TamR and LTED cells compared with those in MCF-7 cells. Let-7 family miRNAs have been generally considered as tumor suppressor miRNAs and the expressions of let-7 family members were reported to be downregulated in many cancer types including breast cancer and at times during tumor progression^43^,^44^. Let-7a and let-7f expression was inversely correlated with that of LIN28, which inhibits the biogenesis of the let-7 family and is shown to contribute to breast tumorigenesis^45^,^46^.

miR-378a-3p was another abundantly and differentially expressed miRNAs among these cells, as its expression in TamR and LTED cells was downregulated compared with that in MCF-7 cells. miR-378a-3p may contribute to the pathophysiology of cancers, which might be suggested by some studies in colorectal cancer and rhabdomyosarcoma^47^,^48^. We further studied miR-378a-3p because its functional role has not been yet elucidated in breast cancer.

### Knockdown of miR-378a-3p reverses tamoxifen-dependent suppression of cell growth in MCF-7 cells

**Figure 2:**
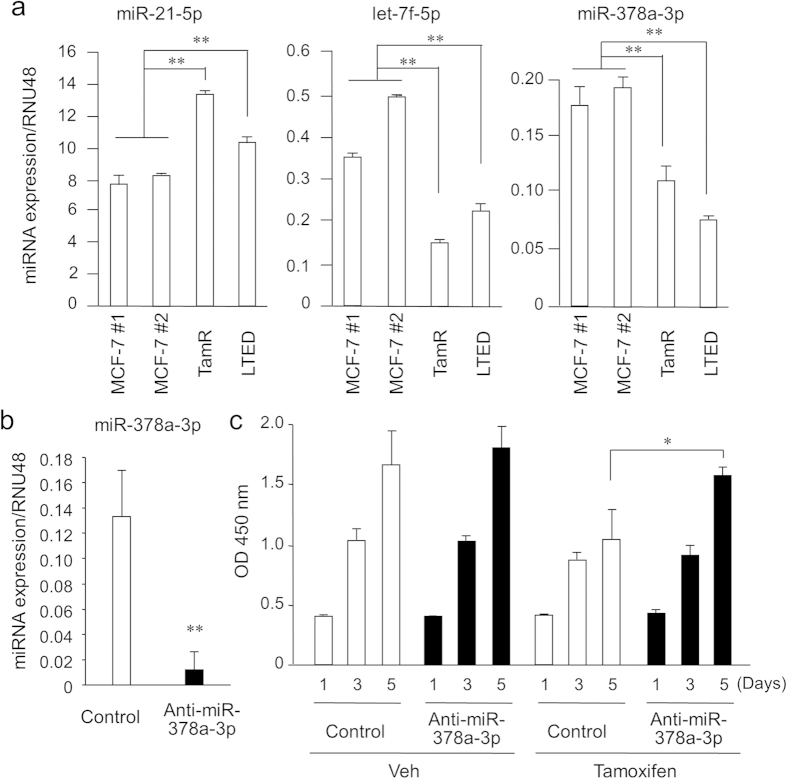
miR-21, let-7f, and miR-378a-3p expression determined by qPCR and effect of miR-378a-3p knockdown on MCF-7 cell growth in the presence of tamoxifen. (**a**) miR-21, let-7f, and miR-378a-3p expression levels in MCF-7, TamR, and LTED cells were determined by qPCR and normalized to RNU48 levels. Data are presented as mean ± SD. Statistical analysis was performed using the Mann-Whitney U test. **P < 0.01. (**b**) Knockdown efficiency of anti-miR-378a-3p. MCF-7 cells were transfected with anti-miR-378a-3p or negative control for 48 h. miR-378a-3p levels were determined by qPCR and normalized to RNU48 levels. Data are presented as mean ± SD in triplicates; **P < 0.01. (**c**) Knockdown of miR-378a-3p significantly increased MCF-7 cell growth in the presence of tamoxifen. Cells were transfected with anti-miR-378a-3p or negative control for 12 h, and then treated with 1 μM tamoxifen or vehicle. Cell viability was analyzed using the WST-8 cell proliferation assay at 1, 3, and 5 days after transfection. Data are presented as mean ± SD, in triplicate; *P < 0.05.

To validate the endogenous expression levels of miR-21-5p, let-7f-5p, and miR-378a-3p, we performed quantitative polymerase chain reaction (qPCR) using cDNAs reversely transcribed from the RNAs prepared from MCF-7, TamR, and LTED cells (Fig. 2a). miR-21 expression levels were significantly upregulated, whereas those of let-7f and miR-378a-3p were significantly downregulated in TamR, and LTED cells compared with MCF-7 cells. To further assess the functional role of miR-378a-3p in the proliferation of breast cancer cells, we performed loss-of-function studies using the miRNA inhibitor, anti-miR-378a-3p. The expression of miR-378a-3p was significantly reduced in MCF-7 cells transfected with anti-miR-378a-3p (Fig. 2b). A cell viability assay showed that tamoxifen treatment significantly repressed the growth of MCF-7 cells transfected with a control miRNA inhibitor. When the cells were transfected with anti-miR-378a-3p, however, tamoxifen-mediated suppression of cell growth was impaired in MCF-7 cells (Fig. 2c). On the other hand, overexpression of miR-378a-3p reduced tamoxifen-resistant growth of TamR cells (Supplementary Fig. S1). These results indicate that miR-378a-3p downregulation will contribute to tamoxifen resistance in breast cancer cells. To assess the downregulation mechanism of miR-378a-3p in TamR cells, we investigated the effect of 5-aza-2′-deoxycytidine (5Aza-dC), an inhibitor of DNA methylation, on the expression of miR-378a-3p (Supplementary Fig. S2). We observed that the expression of miR-378a-3p was increased by the treatment with 5-Aza-dC. Notably, in parental MCF-7 cells, 5Aza-dC was not influential on the expression levels of miR-378a-3p, suggesting that the epigenetic regulation of miR-378a-3p expression would be accompanied with acquired tamoxifen resistance (Supplementary Fig. S3).

### Decreased miR-378a-3p expression in clinical breast cancer tissues is associated with poor prognosis

**Figure 3:**
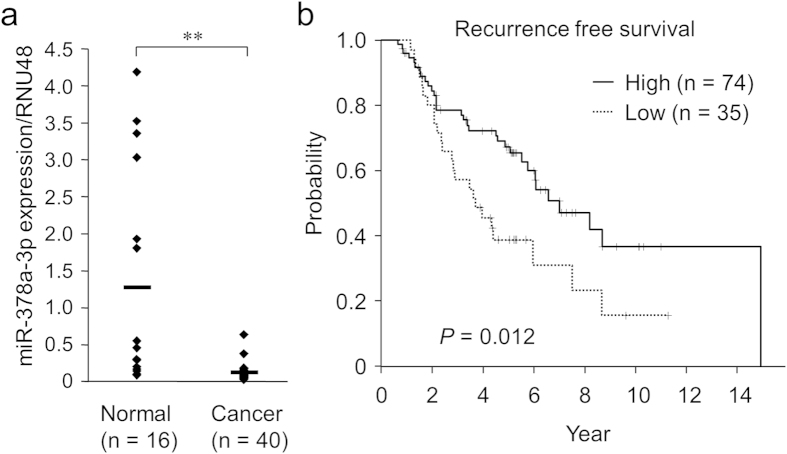
Reduced expression of miR-378a-3p in clinical breast cancer tissues is associated with poor prognosis for tamoxifen-treated patients. (**a**) Decreased miR-378a-3p expression levels in breast cancer tissues compared with those in paired adjacent normal tissues. miR-378a-3p expression levels were determined by qPCR and normalized to RNU48 levels. **P < 0.01. (**b**) Kaplan-Meier survival analysis according to miR-378a-3p levels in tamoxifen-treated breast cancer patients. The recurrence free survival of miR-378a-3p high-expressing group (n = 74) was significantly lower than that of the low-expressing group (n = 35; log-rank test; *P* = 0.012).

miR-378a-3p expression levels were examined in clinical breast cancer samples and adjacent normal tissues (Fig. 3a). miR-378a-3p expression was significantly lower in breast cancer tissues than in adjacent normal tissues. Moreover, we assessed the association between miR-378a-3p levels and clinical outcomes using a breast cancer cohort with previously reported miRNA microarray data deposited in the Gene Expression Omnibus (GEO) database (GSE37405)(Fig. 3b). This dataset contains the miRNA expression data of 152 ER-positive primary breast cancers from high-risk patients. All patients had received adjuvant tamoxifen therapy as a monotherapy (median clinical follow-up: 4.6 years) and the half of the patients had developed distant recurrence (median time-to-recurrence: 3.5 years)^49^. Using the data of 109 patients out of 152 patients for whom miR-378a-3p expression data were available, Kaplan-Meier analysis was performed to evaluate survival (time to recurrence) for groups with high and low expression of miR-378a-3p. Kaplan-Meier survival analysis indicates that patients with low miR-378a-3p levels presented poor recurrence-free survival (log-rank, *P* = 0.012) (Fig. 3b). The results indicate that miR-378a-3p downregulation could be correlated with the development and progression of tamoxifen resistance in breast cancer.

### Identification of candidate target genes for miR-378a-3p

**Figure 4:**
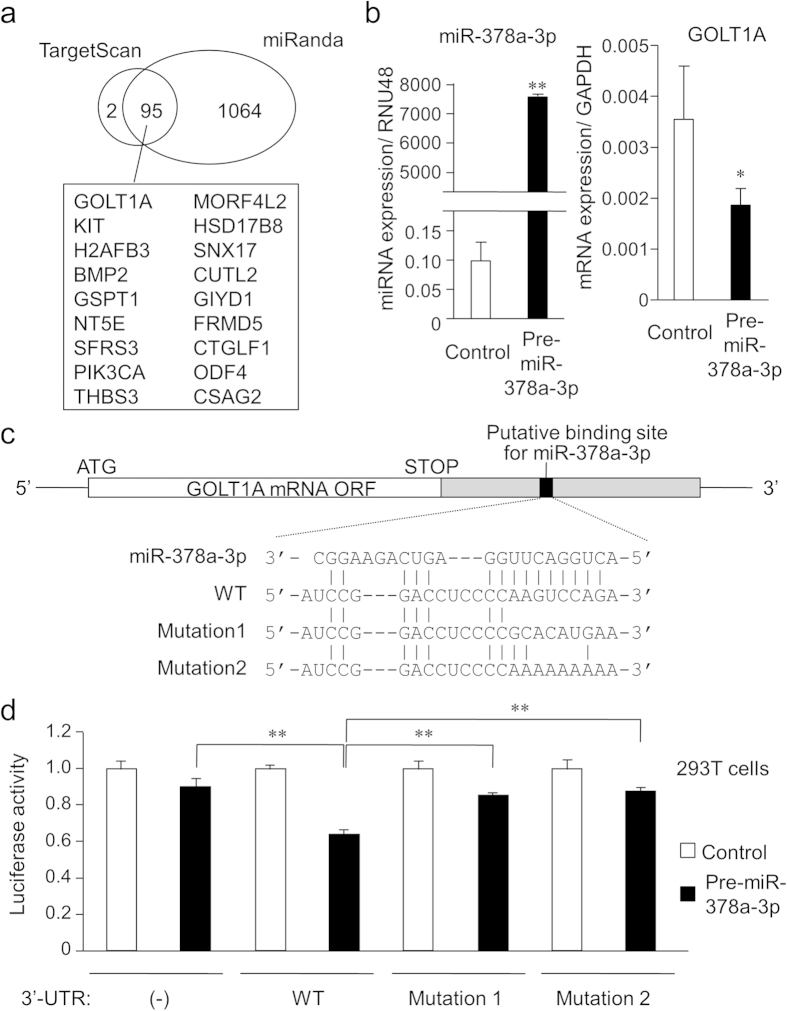
Identification of miR-378a-3p target genes in breast cancer (**a**) Schematic presentation of miR-378a-3p target prediction by *in silico* analyses. Venn diagrams indicating numbers of candidate hits determined by the TargetScan and miRanda prediction algorithms. Candidate genes commonly predicted by the algorithms are described. (**b**) Overexpression of miR-378a-3p suppressed *GOLT1A* mRNA expression. MCF-7 cells were transfected with pre-miR-378a-3p or control miR for 48 h and the expression levels of miR-378a-3p and *GOLT1A* mRNA were then evaluated by qPCR and qRT-PCR, respectively. Data are presented as mean ± SD; *P < 0.05; **P < 0.01. (**c**) Location of putative miR-378a-3p-binding sequence in the 3′-UTR of *GOLT1A* gene and its mutated sequences in luciferase reporters. (**d**) Luciferase reporter assay using vectors containing a putative GOLT1A 3′-UTR binding site for miR-378a-3p and mutated versions of the site. MCF-7 cells were transiently transfected with psiCHECK2 vectors containing either the wild-type or mutated putative binding sites for miR-574-3p, together with pre-miR-378a-3p precursor or control pre-miR for 48 h. The luciferase assay was then performed. Data are presented as mean ± SD; **P < 0.01.

To identify miR-378a-3p candidate targets, we used 2 target gene prediction programs, TargetScan^50^ and miRanda^51^ (Fig. 4a). TargetScan and miRanda identified 97 and 1,159 candidate targets for miR-378a-3p, respectively. Notably, 95 genes were common between these programs. Of these candidates, we focused on *GOLT1A* because it presented one of the highest precision score. To examine whether miR-378a-3p directly regulates *GOLT1A* expression, we transfected MCF-7 cells with pre-miR-378a-3p for 48 h and then evaluated *GOLT1A* mRNA expression by quantitative reverse transcription polymerase chain reaction (qRT-PCR). Transfection with pre-miR-378a-3p significantly decreased *GOLT1A* mRNA expression level in MCF-7 cells compared to that of the control pre-miR (Fig. 4b). We next constructed luciferase reporter vectors containing either the wild-type *GOLT1A* 3′-UTR sequence with a putative binding site for miR-378a-3p or the altered sequences of the 3′-UTR in which the putative binding site was mutated (mutations 1 and 2) (Fig. 4c). In 293T cells, the luciferase assay demonstrated that miR-378a-3p decreased the activity of luciferase reporter with the wild-type sequence, but not that of the reporters with the mutated sequences (Fig. 4d). This result suggests that miR-378a-3p regulates *GOLT1A* expression by binding to the 3′-UTR of *GOLT1A*.

### GOLT1A knockdown restores tamoxifen sensitivity and low GOLT1A levels are associated with better survival in patients with breast cancer

**Figure 5:**
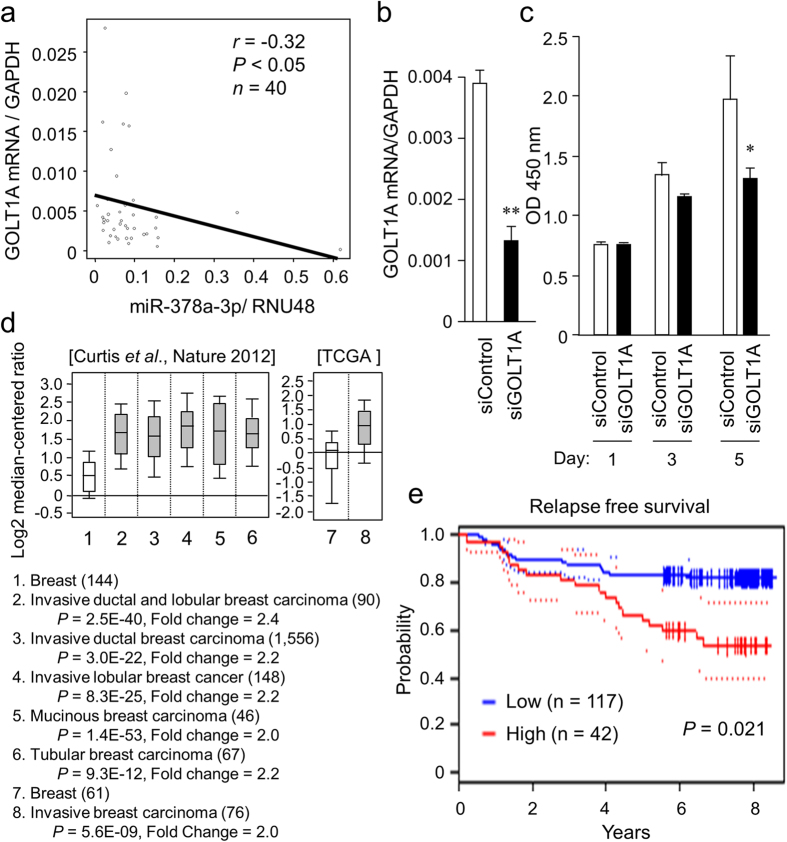
GOLT1A knockdown restored tamoxifen resistance and low *GOLT1A* levels correlate with better survival in patients with breast cancer. (**a**) Negative correlation between miR-378a-3p and *GOLT1A* expression levels in breast cancer tissues. The expression levels of miR-378a-3p and *GOLT1A* mRNA were examined in cancer samples used in Fig. 3a. (**b**) Knockdown efficiency of GOLT1A siRNA. TamR cells were transfected with siGOLT1A or negative control for 48 h. *GOLT1A* mRNA levels were determined by qRT-PCR and normalized to *GAPDH* levels. Data are presented as mean ± SD, in triplicate; **P < 0.01. (**c**) GOLT1A knockdown significantly reduced TamR cell growth in the presence of tamoxifen. Cells were transfected with siGOLT1A or negative control. Cell viability was then analyzed by WST-8 cell proliferation assay at 1, 3, and 5 days after transfection. Data are presented as mean ± SD, in triplicate; *P < 0.05. (**d**) *GOLT1A* mRNA was overexpressed in clinical breast cancer tissues compared to normal mammary tissues, based on the Oncomine cancer profiling database, by >2-fold at *P* < 1e-8. (**e**) *GOLT1A* expression correlates with poor relapse-free survival in patients with breast cancer. Survival curves for high (n = 42) and low (n = 117) expression groups dichotomized at the optimal cut point are plotted using the PrognoScan^64^. The 95% confidence intervals for each group are also indicated by dotted lines.

Since GOLT1A was shown as a candidate miR-378a-3p target in breast cancer cells, we investigated whether a negative correlation between miR-378a-3p and *GOLT1A* expression can be observed in clinical breast cancer specimens (Fig. 5a). A negative correlation was shown between the expression levels of miR-378a-3p and *GOLT1A* (*r* = −0.32, *P* < 0.05). To examine a functional role of GOLT1A in the tamoxifen response, we performed a loss-of-function study in TamR cells using *GOLT1A* siRNA. We found that *GOLT1A* mRNA expression was markedly repressed in TamR cells transfected with *GOLT1A* siRNA (Fig. 5b). Cell proliferation assay showed a significant growth inhibition of cells transfected with *GOLT1A* siRNA as compared to the control transfectants (Fig. 5c). It was confirmed by using another siRNA (siGOLT1A-2) that the growth inhibitory effect of GOLT1A silencing was observed markedly when TamR cells were treated with tamoxifen (Supplementary Fig. S4). To further examine whether the tumor-suppressive effect of miR-378a-3p is mediated by GOLT1A, MCF-7 cells were transfected with the combinations of anti-miR-378a-3p and siGOLT1A (Supplementary Fig. S5). Anti-miR-378a-3p transfection upregulated the MCF-7 cell growth in the presence of tamoxifen compared with anti-miR negative control transfection; however, the tamoxifen-resistant growth induced by anti-miR-378a-3p was abrogated by siGOLT1A. We then examined the Oncomine microarray database to determine whether *GOLT1A* expression was altered in other cohorts of clinical breast cancer samples^52^. Two datasets indicated that *GOLT1A* was substantially overexpressed (by > 2-fold) in clinical breast cancers compared to normal breast tissues (*P* < 1e-8, Fig. 5d). Of note, we found that low *GOLT1A* expression was associated with good prognosis for breast cancer in a breast cancer microarray dataset (Fig. 5e). These results suggest that *GOLT1A* plays a role in malignant alteration of breast cancer, including the acquisition of tamoxifen resistance.

## Discussion

In the present study, we identified miRNAs that are differentially expressed in endocrine therapy resistant breast cancer cells (TamR and LTED) compared to parental MCF-7 cells. Among them, miR-21 and let-7 (let-7f and let-7a) that are generally known as an onco-miRNA and an anti-onco-miRNA, respectively, were the top three differentially expressed miRNAs in the endocrine therapy resistant breast cancer cells. miR-21 and let-7s (let-7f and let-7a) expression levels are increased and decreased, respectively, in the endocrine therapy resistant breast cancer cells, suggesting their roles that will contribute to aggressive type of breast cancer, including the acquisition of tamoxifen resistance^35^,^43^.

As the fourth most differentially expressed miRNA in the endocrine therapy resistant breast cancer cells, we focused on miR-378a-3p whose expression levels were decreased in these cells. miR-378a-3p knockdown facilitated MCF-7 cell growth in the presence of tamoxifen. In addition, miR-378a-3p expression was reduced in clinical breast cancer tissues compared with cancer-surrounding normal tissues and low expression of miR-378a-3p was associated with poor prognosis in ER-positive breast cancer patients treated with adjuvant tamoxifen. In a clinicopathological study of colorectal cancer, miR-378a-3p expression is decreased in cancer tissues compared to that in adjacent normal colorectal tissues^47^. miR-378a-3p expression was correlated with pathological parameters, including histological differentiation and TNM stage, and low miR-378a-3p expression was significantly associated with shorter survival time. Ectopic miR-378a-3p expression could inhibit colorectal cancer cell growth and colony formation and induce apoptosis and cell cycle arrest. Loss- and gain-of-function experiments of miR-378a-3p in colorectal cancer cells suggest that miR-378a-3p may regulate the expression of the insulin-like growth factor 1 receptor (*IGF1R*) as a target gene. A significant negative correlation between IGF1R protein and miR-378a-3p expression levels was observed in colorectal cancer tissues. In another study, miR-378a-3p downregulation is observed in rhabdomyosarcoma tissues and cell lines^48^. miR-378a-3p overexpression suppressed IGF1R expression in rhabdomyosarcoma-derived RH30 cells and decreased the phosphorylation level of AKT protein, which is known as a key signaling molecule in rhabdomyosarcoma. miR-378a-3p overexpression resulted in an increase of apoptosis and a decrease of cell growth in RH30 cells. miR-378a-3p could also induce myogenic differentiation by modulating myogenic marker genes in RH30 cells. In this report, treatment with the DNA methyltransferase inhibitor, 5-Aza-dC, upregulated miR-378a-3p expression. These findings together with our data suggest that miR-378a-3p may play a tumor-suppressive role in malignant tumors. In breast cancer, we assumed that miR-378a-3p downregulation may particularly contribute to the acquisition of endocrine therapy resistance. Epigenetic modification may lead to the deregulation of miR-378a-3p expression in breast cancer cells^53^,^54^.

We selected *GOLT1A* as one of the miR-378a-3p candidate target genes by *in silico* algorithms. Our computational analysis is consistent with our experimental results that *GOLT1A* mRNA level was downregulated in MCF-7 cells transfected with miR-378a-3p precursor. Luciferase reporter assay using the 3′-UTR region of GOLT1A gene including a putative binding site for miR-378a-3p suggested that miR-378a-3p could decrease *GOLT1A* mRNA expression. In addition, GOLT1A knockdown by siRNA restored tamoxifen sensitivity in TamR cells. Moreover, the examination of publicly available microarray datasets revealed that high levels of *GOLT1A* expression correlated with poor rates of survival for patients with breast cancer. These results suggest that miR-378a-3p and its candidate target gene, *GOLT1A*, could contribute to the mechanisms underlying the endocrine resistance of breast cancer.

GOLT1A is an evolutionarily conserved protein with a structure including four putative transmembrane domains. Got1p has been identified as a yeast homolog of GOLT1A by screening for mutations that show synthetic lethality with a membrane protein sft2, which is localized in the Golgi apparatus^55^. The yeast study showed that Got1p protein is present in the early Golgi cisternae. Got1p mutant yeast exhibited reduced endoplasmic reticulum (ER)-to-Golgi transport due to the impairment of SNARE-dependent fusion. Another yeast study revealed that Got1p displayed moderate suppressor activity toward temperature-sensitive mutations in the *SEC23* and *SEC31* genes, which encode subunits of the coat protein complex II (COPII), a type of *vesicle* coat protein that transports forward from the rough ER to the Golgi apparatus^56^. The study further showed that Got1p was efficiently packaged into COPII vesicles and cycled rapidly between the ER and Golgi compartments. As overexpression of yeast Got1p or N-terminal region of human GOLT1A rather impaired the ER-to-Golgi transport^56^,^57^, GOLT1A could plausibly saturate or somehow interfere the normal cargo recongnition functions of the COPII vesicles. Further studies will be required to fully assess the role of GOLT1A in the ER-to-Golgi network with differential expression levels, nevertheless, GOLT1A could influence membrane properties and modulate vesicle formation in the ER-to-Golgi network.

Recent evidence suggests that the ER-to-Golgi network regulates cellular processes including stress response, apoptosis, and mitotic checkpoint in cancer cells. In prostate cancer, Golgi is emerging as a new therapeutic target since several Golgi-associated molecules have been demonstrated to be regulated by androgen^58^. Brefeldin A (BFA) is known as is an inhibitor of ER-to-Golgi protein transport by inhibiting the interaction between ADP-ribosylation factor 1 (Arf1) and guanine nucleotide exchange factor (GEF). Treatment of mammalian cells with BFA induces both ER and Golgi stress due to its ability to cause a cytotoxic accumulation of proteins in the ER that would normally be trafficked through the Golgi to be processed for secretion. The stress to both organelles resulting from BFA treatment can induce apoptosis^59^,^60^. The inhibition of the Arf1-GEF interaction will be beneficial for cancer therapy. For example, limited effective therapeutic options are available for patients with chronic lymphocytic leukemia (CLL) refractory to alkylating agents such as fludarabine^61^. It is notable that BFA could induce apoptosis in primary CLL cells from fludarabine-refractory patients^61^. Although BFA and its derivatives have not progressed beyond the pre-clinical stage of drug development because of their poor bioavailability, targeting the ER-to-Golgi network will be an attractive therapeutic option for cancers, especially for those that respond poorly to conventional treatments. It remains to be investigated whether new inhibitors for the ER-to-Golgi network can manage tamoxifen-resistant breast cancers, particularly those with high expression of GOLT1A and with altered ER-to-Golgi network.

In summary, we have identified miR-378a-3p as a modulating factor for the tamoxifen response in breast cancer by high throughput sequencing. A combination of *in silico* and *in vitro* analyses indicates that GOLT1A is a potential target of miR-378a-3p. These findings could be applied to develop alternative approaches to breast cancer diagnosis and treatment.

## Methods

### Cell culture

MCF-7 and 293T cells were purchased from ATCC (Manassas, VA, USA) and cultured in Dulbecco’s modified Eagle’s Medium (DMEM) supplemented with 10% fetal bovine serum (FBS), 50 units/ml penicillin, and 50 μg/ml streptomycin at 37 °C in a humidified atmosphere of 5% CO_2_ in air. Resistant clones for tamoxifen (TamR) were established from MCF-7 cells by long-term (>3 months) culture with 1 μM tamoxifen^32^. Tamoxifen was purchased from Sigma (St. Louis, MO, USA). Long-term estrogen-deprived (LTED) cells were established from MCF-7 cells by culturing them in phenol red-free DMEM supplemented with 10% dextran-charcoal stripped FBS (dccFBS).

### RNA extraction and high-throughput sequencing

Total RNA was isolated from MCF-7, TamR and LTED cells using the ISOGEN reagent (Nippon Gene, Toyama, Japan) in accordance with the manufacturer’s instructions. Small RNA cDNA library was generated from the total RNAs and high-throughput sequencing was performed using an Illumina GAIIx sequencer (Illumina, San Diego, CA, USA)^62^. Mapping of small RNA reads on human genomes (NCBI35 assembly) and prediction of novel miRNAs were performed as describes previously^34^.

### Transfection of miRNA precursors/inhibitors and siRNA

Pre-miR-378a-3p and its positive control as well as anti-miR-378a-3p and its negative control were purchased from Ambion (Carlsbad, CA, USA). Transfection of miRNA precursors or inhibitors was carried out using Lipofectamine RNAiMAX transfection reagent (Invitrogen, Carlsbad, CA, USA) according to the manufacturer’s instruction. siRNA duplexes, siGOLT1A (5′-GAUUCUUCAGCCUCUUUAAGG-3′ and 5′-UUAAAGAGGCUGAAGAAUCCG-3′) and siGOLT1A-2 (5′-GAAACCUACGGAUUCUUCA-3′ and 5′-UGAAGAAUCCGUAGGUUUCCA-3′) both of which target GOLT1A was synthesized using an algorithm that significantly improves the target specificity of siRNA, in particular, by efficiently estimating off-target sequences^63^. A non-targeting control siRNA (siControl) with no homology to known gene targets in mammalian cells was obtained from RNAi Inc. (Tokyo, Japan)^63^. Cells were cultured overnight and transfected with siRNA at a final concentration of 10 nM using Lipofectamine RNAiMAX. Knockdown efficiency of siRNA was determined by qRT-PCR using RNA prepared from the cells 48 h after transfection and normalized to that of siControl.

### qPCR and qRT-PCR

Total RNA was extracted from cells or tissues using the ISOGEN reagent. For treatment of 5-aza-2′-deoxycytidine (5-Aza-dC), cells were incubated with 1 or 10 μM 5-Aza-dC or vehicle for 72 h. miRNA levels were determined by qPCR using triplicate TaqMan microRNA assays (Applied Biosystems, CA, USA). The target gene mRNA levels were evaluated by the StepOne Real-time PCR System (Applied Biosystems) using cDNAs converted from total RNA with SuperScript III Reverse Transcriptase (Invitrogen). Results from 3 independent experiments were normalized to the expression of endogenous RNU48 for miRNA or that of GAPDH for mRNA, respectively. Primers for GOLT1A and GAPDH were as follows: GOLT1A forward: 5′- GGGCCTGTCCCTCATCATT -3′,

GOLT1A reverse: 5′- TTTGTGCCGTTGGAAGAAGAA -3′,

GAPDH forward: 5′-GGTGGTCTCCTCTGACTTCAACA-3′, and

GAPDH reverse: 5′-GTGGTCGTTGAGGGCAATG-3′.

### miRNA target prediction

Candidate targeted genes by miR-378a-3p were examined using 2 online database algorithms for miRNA target prediction: TargetScan (http://www.targetscan.org/) and miRanda (http://www.microrna.org/microrna/getGeneForm.do)^50^,^51^.

### Cell growth assay

Cell proliferation was evaluated using the WST-8 assay kit (Nacalai Tesque, Kyoto, Japan). Two thousands of MCF-7 or TamR cells per well were cultured in 96-well plates and 10 μL of WST-8 solution was added to each well at the indicated time points after transfection. Cells were further incubated for 2 h at 37 °C in a 5% CO_2_ incubator. The absorbance was measured at 450 nm with Multiscan FC Microplate Photometer (Thermo Fisher Scientific, Rochester, NY, USA).

### Luciferase reporter assay

The sequence of GOLT1A 3′-UTR containing a putative binding site for miR-378a-3p was amplified by specific primers using MCF-7 cDNA and inserted into the psiCHECK-2 vector (Promega, Madison, WI, USA). Two mutants for the putative binding site for miR-378a-3p were constructed by PCR with the mutated primers. For 3′-UTR luciferase assay, 293T cells were transfected with psiCHECK2 vector containing the wild-type or mutated putative binding sites for miR-378a-3p together with pre-miR-378a-3p precursor or control pre-miR using Lipofectamine 2000 transfection reagent (Invitrogen). The psiCHECK2 empty vector was also transfected as a mock control. Luciferase reporter assay was performed using the Dual-Luciferase Reporter Assay System (Promega) 48 hours after transfection. Firefly luciferase activities for GOLT1A 3-UTR reporters were normalized to those for corresponding Renilla luciferase activities. Experiments were performed in triplicate, and the results were expressed as mean ± SD.

### Clinical specimens

All clinical breast cancer tissues (n = 40) and the adjacent normal tissues (n = 16) were resected from patients those received surgery at Saitama Medical University. All procedures were performed under a protocol approved by the institutional Ethics Committee, and written informed consent was obtained from all patients. The methods were carried out in accordance with the approved guidelines. Total RNA was isolated from these dissected samples and subjected to qPCR and qRT-PCR analyses. Oncomine Research Edition^46^ was used for the evaluation of *GOLT1A* mRNA expression in clinical breast cancer and normal mammary tissues based on microarray datasets. Kaplan–Meier curves of relapse-free survival times stratifying breast cancer patients by GOLT1A expression were obtained using the Prognoscan (http://www.abren.net/PrognoScan/), which is a database for meta-analysis of the prognostic value of genes^64^.

We also used the publicly available dataset GSE37405^49^ from the Gene Expression Omnibus repository (http://www.ncbi.nlm.nih.gov/geo/query/acc.cgi? acc = GSE37405), which contains miRNA expression data of ER-positive breast cancer patients treated with adjuvant tamoxifen. Kaplan–Meier analysis of recurrence-free survival was performed using a statistical software package EZR (Saitama Medical Center, Jichi Medical University, Saitama, Japan)^65^.

## Additional Information

**How to cite this article**: Ikeda, K. *et al.* miR-378a-3p modulates tamoxifen sensitivity in breast cancer MCF-7 cells through targeting *GOLT1A*. *Sci. Rep.* **5**, 13170; doi: 10.1038/srep13170 (2015).

## Supplementary Material

Supplementary Information
