# Supplementary material for: miR-378a-3p modulates tamoxifen sensitivity in breast cancer MCF-7 cells through targeting GOLT1A
Source: Sci Rep. 2015 Aug 10;5:13170. doi: 10.1038/srep13170 (PMC4530347; doi:10.1038/srep13170)
Supplement: Supplementary Information [file srep13170-s1.pdf]

miR-378a-3p modulates tamoxifen sensitivity in breast cancer MCF-7 cells through targeting *GOLT1A*

Kazuhiro Ikeda, Kuniko Horie-Inoue, Toshihide Ueno, Takashi Suzuki, Wataru Sato, Takashi Shigekawa, Akihiko Osaki, Toshiaki Saeki, Eugene Berezikov, Hiroyuki Mano, Satoshi Inoue

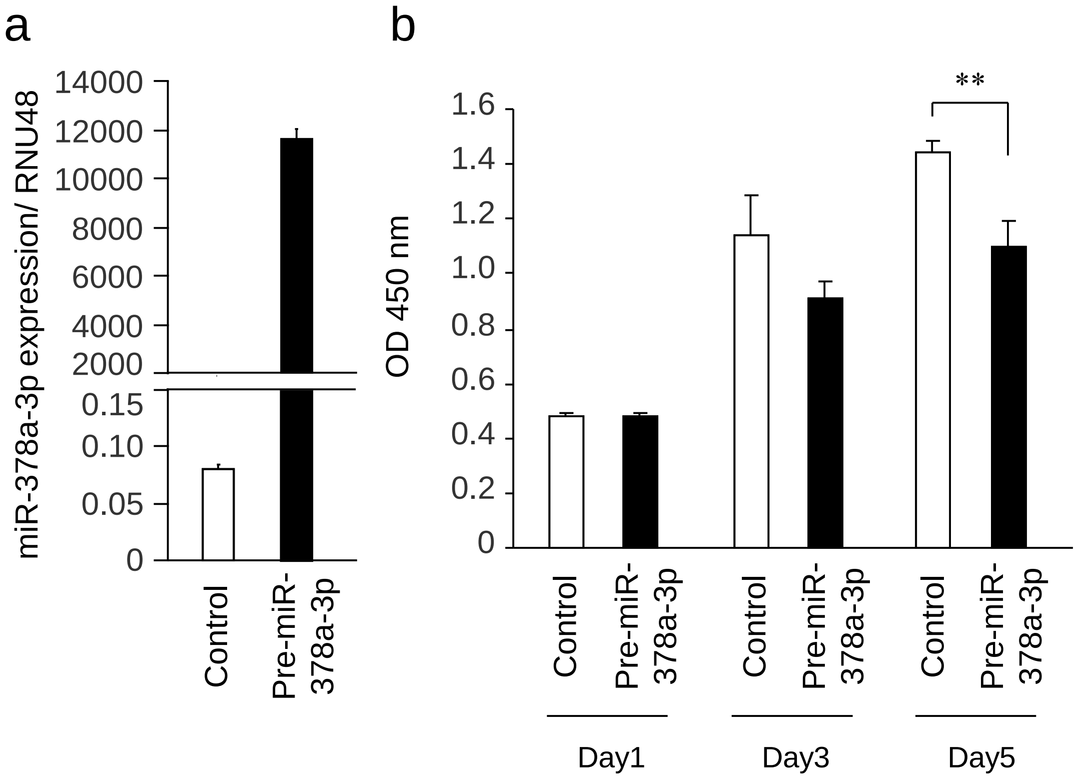

Supplementary Figure S1. miR-378a-3p reduced TamR cell growth. Cells were transfected with **pre-**miR-378a-3p or control, and treated with 1 μM tamoxifen. Cell viability was analyzed by WST-8 cell proliferation assay at 1, 3, and 5 days after transfection. Data are presented as mean ± s.d. (n = 3; **, *P* < 0.01).

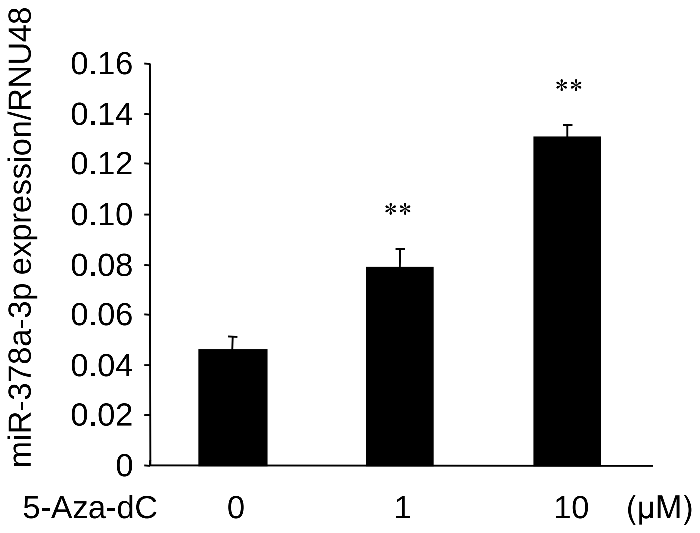

Supplementary Figure S2. DNA methyltransferase inhibitor 5-aza-2'-deoxycytidine upregulates miR-378a-3p expression in TamR cells. Cells were treated with 1 or 10 μM 5-aza-2'-deoxycytidine (5-Aza-dC) or vehicle for 72 h. miR378a-3p and RNU48 levels were analyzed by qPCR, performed in triplicate. miR-378a-3p expression was normalized to RNU48 and shown as mean ± s.d. (n = 3; **, $P < 0.01$).

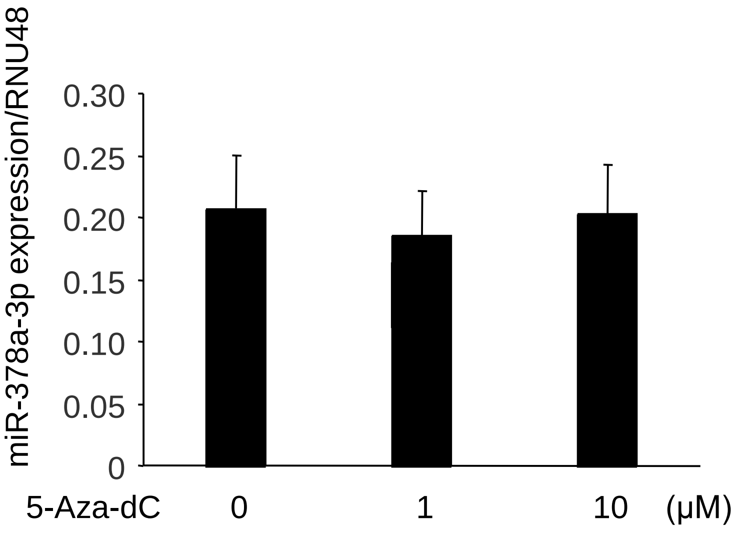

Supplementary Figure S3. miR-378a-3p expression is not influenced by treatment of 5-aza-2'-deoxycytidine in parental MCF-7 cells. Cells were treated with 1 or 10 μM 5-aza-2'-deoxycytidine (5-Aza-dC) or vehicle for 72 h. miR378a-3p and RNU48 levels were analyzed by qPCR, performed in triplicate. miR-378a-3p expression was normalized to RNU48 and shown as mean ± s.d. (n = 3; **, $P < 0.01$).

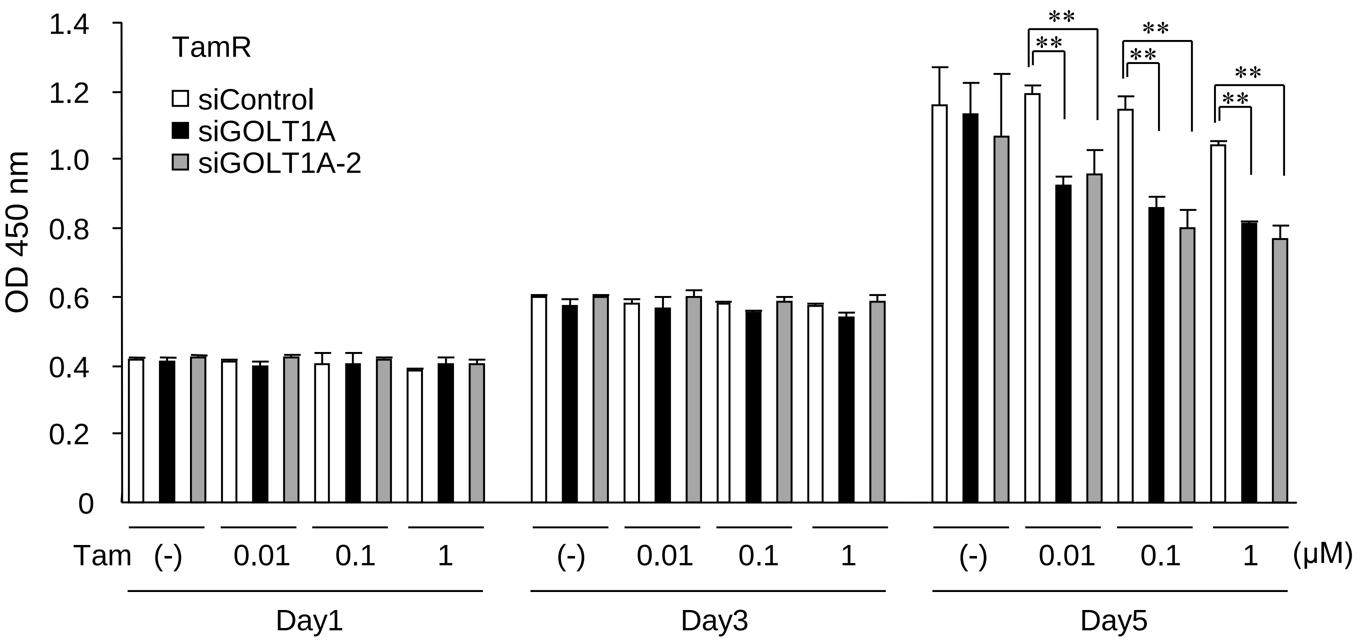

Supplementary Figure S4. GOLT1A knockdown reduced TamR cell growth dependent on the presence of tamoxifen. Cells were transfected with siGOLT1A, siGOLT1A-2 or negative control, and treated with 0.01, 0.1, or 1 μM tamoxifen (Tam) or vehicle (-). Cell viability was analyzed by WST-8 cell proliferation assay at 1, 3, and 5 days after transfection. Data are presented as mean ± s.d. (n = 3; **, $P < 0.01$).

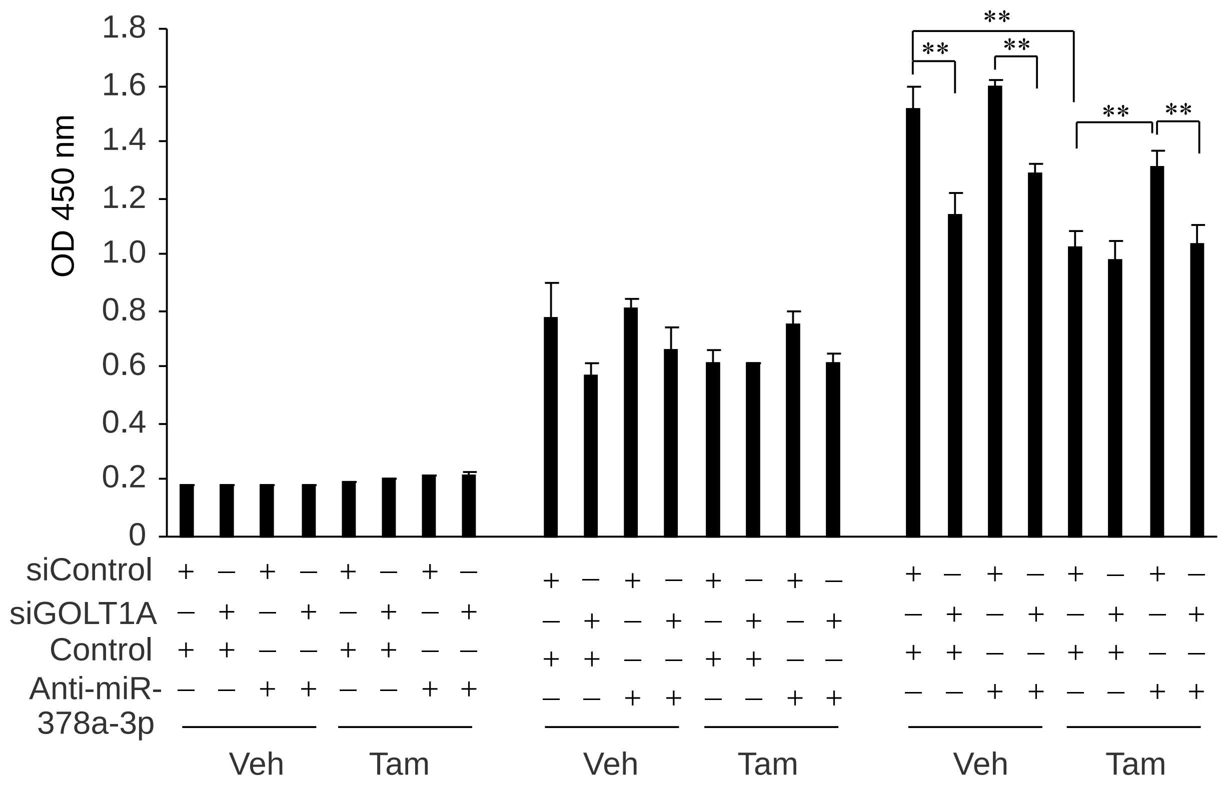

Supplementary Figure S5. Increased cell growth by miR-378a-3p knockdown is abrogated by GOLT1A silencing in TamR cells. Cells were transfected with the combinations of siGOLT1A or negative control siRNA (siControl) and anti-miR-378a-3p or anti-miR negative control (Control), and treated with or without 1 μM tamoxifen. Cell viability was analyzed by WST-8 cell proliferation assay at 1, 3, and 5 days after transfection. Data are presented as mean ± s.d. (n = 3; **, *P* < 0.01).

Supplementary Table S1. List of annotated miRNAs in MCF-7, TamR, and LTED cells.

**miR**	**MCF-7 #1** (%)	**MCF-7 #2** (%)	**TamR** (%)	**LTED** (%)	**Fold change: TamR vs MCF-7s**	**Fold change: LTED vs MCF-7s**
hsa-mir-21-5p	61.4300	66.0780	78.9400	80.1174	1.2382	1.1049
hsa-let-7f-5p	8.9411	7.2077	4.6630	5.8129	0.5775	0.9794
hsa-let-7a-5p	4.9703	3.9738	2.2483	1.9738	0.5027	0.6344
hsa-mir-378a-3p	2.6922	2.2412	1.5110	0.8959	0.6126	0.4776
hsa-mir-200c-3p	3.0978	2.9198	1.0711	0.6561	0.3560	0.3288
hsa-mir-30d-5p	1.1743	1.2033	0.9033	0.7976	0.7599	0.7573
hsa-mir-182-5p	1.2368	1.0389	0.8370	0.4590	0.7356	0.4894
hsa-mir-27b-3p	0.7151	0.8168	0.7877	0.4892	1.0284	0.6098
hsa-mir-99b-5p	0.7329	0.8050	0.7696	0.2982	1.0009	0.3787
hsa-mir-24-3p	1.3136	1.0908	0.6134	0.8550	0.5102	1.0034
hsa-mir-101-3p	0.7769	0.8532	0.5119	0.2997	0.6281	0.4390
hsa-mir-103a-3p	0.8779	0.7412	0.4942	0.3901	0.6105	0.6316
hsa-mir-26a-5p	0.4521	0.3619	0.3978	0.2717	0.9773	0.7153
hsa-mir-93-5p	0.4990	0.5775	0.3680	0.2162	0.6838	0.4572
hsa-mir-141-3p	0.7367	0.6522	0.3200	1.1778	0.4608	2.4229
hsa-mir-148a-3p	0.7137	0.6139	0.3152	0.0634	0.4749	0.1364
hsa-mir-183-5p	0.5758	0.4921	0.2993	0.1804	0.5605	0.4560
hsa-mir-27a-3p	0.2959	0.3357	0.2920	0.2332	0.9247	0.7432
hsa-mir-374a-3p	0.3757	0.4140	0.2586	0.1681	0.6549	0.4999
hsa-mir-30e-5p	0.3049	0.2548	0.2569	0.5188	0.9180	2.0277
hsa-mir-151a-3p	0.3587	0.3320	0.2477	0.1941	0.7171	0.6697
hsa-mir-200b-3p	0.0988	0.0634	0.2113	0.1272	2.6045	0.9260
hsa-mir-203-3p	0.2662	0.2596	0.1947	0.3034	0.7408	1.3356
hsa-let-7d-5p	0.3774	0.3522	0.1766	0.0697	0.4841	0.2635
hsa-mir-23b-3p	0.2310	0.1801	0.1615	0.0541	0.7855	0.3169
hsa-mir-106b-5p	0.2808	0.3480	0.1499	0.1547	0.4769	0.6213
hsa-mir-425-5p	0.7521	0.6384	0.1441	0.1720	0.2073	0.4395
hsa-mir-148b-3p	0.3917	0.3103	0.1414	0.0479	0.4028	0.2120
hsa-mir-200a-3p	0.0569	0.0654	0.1355	0.0826	2.2169	0.8222
hsa-let-7b-5p	0.2518	0.2076	0.1315	0.2827	0.5725	1.6673
hsa-mir-140-3p	0.1365	0.1370	0.1221	0.0697	0.8932	0.5383

Supplementary Table S1. (continued)

miR	MCF-7 #1 (%)	MCF-7 #2 (%)	TamR (%)	LTED (%)	Fold change: TamR vs MCF-7s	Fold change: LTED vs MCF-7s
hsa-mir-23a-3p	0.1211	0.1423	0.1133	0.0571	0.8605	0.4464
hsa-mir-429-3p	0.0714	0.0474	0.1106	0.0927	1.8617	1.1744
hsa-mir-181a-5p	0.2786	0.2385	0.1078	0.1480	0.4171	0.8548
hsa-let-7g-5p	0.1635	0.1395	0.1023	0.0821	0.6754	0.6792
hsa-let-7e-5p	0.1274	0.1404	0.1015	0.2139	0.7583	1.7688
hsa-mir-191-5p	0.4254	0.3319	0.0979	0.0906	0.2586	0.4216
hsa-mir-32-5p	0.1415	0.0736	0.0946	0.0316	0.8801	0.3755
hsa-mir-125a-5p	0.1123	0.1218	0.0924	0.0535	0.7897	0.4997
hsa-mir-363-3p	0.0554	0.0448	0.0677	0.0003	1.3498	0.0056
hsa-mir-152-3p	0.0764	0.0647	0.0674	0.0236	0.9552	0.3579
hsa-mir-22-3p	0.0352	0.0541	0.0666	0.0063	1.4908	0.1036
hsa-mir-181b-5p	0.1301	0.1404	0.0594	0.0827	0.4394	0.8281
hsa-mir-1307-3p	0.0874	0.1023	0.0528	0.0175	0.5567	0.2261
hsa-mir-340-5p	0.1727	0.0722	0.0516	0.0418	0.4216	0.6755
hsa-mir-26b-5p	0.0628	0.0706	0.0514	0.0981	0.7712	1.6071
hsa-let-7i-5p	0.0803	0.0829	0.0501	0.0993	0.6136	1.4938
hsa-mir-25-3p	0.0718	0.0617	0.0484	0.0484	0.7252	0.8787
hsa-mir-146b-5p	0.0412	0.0448	0.0468	0.0500	1.0869	1.0917
hsa-mir-20a-5p	0.1564	0.1497	0.0462	0.0411	0.3020	0.4193
hsa-mir-542-3p	0.0263	0.0321	0.0418	0.0581	1.4311	1.5706
hsa-mir-126-3p	0.0974	0.0837	0.0407	0.0441	0.4496	0.7089
hsa-mir-16-5p	0.0633	0.0271	0.0391	0.1882	0.8649	5.6922
hsa-mir-21-3p	0.0270	0.0313	0.0374	0.0410	1.2834	1.1928
hsa-mir-98-5p	0.0881	0.0795	0.0352	0.0393	0.4201	0.6857
hsa-mir-744-5p	0.1429	0.1480	0.0341	0.0229	0.2345	0.2514
hsa-mir-15a-5p	0.0697	0.0448	0.0336	0.0336	0.5863	0.8579
hsa-mir-186-5p	0.1159	0.0829	0.0314	0.0208	0.3156	0.3642
hsa-mir-30a-5p	0.4699	0.5623	0.0303	0.0423	0.0586	0.1428
hsa-mir-96-5p	0.0419	0.0287	0.0292	0.0351	0.8250	1.2135
hsa-mir-17-5p	0.1038	0.1074	0.0289	0.0284	0.2735	0.4162
hsa-mir-30c-5p	0.0700	0.0516	0.0281	0.0220	0.4615	0.5535

Supplementary Table S1. (continued)

miR	MCF-7 #1 (%)	MCF-7 #2 (%)	TamR (%)	LTED (%)	Fold change: TamR vs MCF-7s	Fold change: LTED vs MCF-7s
hsa-mir-1307-5p	0.0462	0.0592	0.0270	0.0204	0.5115	0.4735
hsa-mir-92a-3p	0.0668	0.0516	0.0264	0.0184	0.4461	0.4713
hsa-mir-192-5p	0.0348	0.0372	0.0264	0.0213	0.7332	0.6697
hsa-mir-423-3p	0.0540	0.0600	0.0264	0.0323	0.4630	0.7481
hsa-mir-30e-3p	0.0426	0.0389	0.0259	0.0592	0.6342	1.8271
hsa-mir-193b-3p	0.0334	0.0194	0.0259	0.0117	0.9784	0.5146
hsa-mir-769-5p	0.0345	0.0431	0.0220	0.0150	0.5672	0.4616
hsa-mir-30b-5p	0.0172	0.0203	0.0209	0.0088	1.1140	0.4256
hsa-mir-29b-3p	0.1159	0.0364	0.0204	0.1060	0.2674	3.7364
hsa-mir-454-3p	0.0199	0.0127	0.0176	0.0152	1.0805	1.0034
hsa-mir-29a-3p	0.0092	0.0127	0.0165	0.0201	1.5056	1.3774
hsa-mir-301a-3p	0.0171	0.0135	0.0160	0.0253	1.0431	1.7191
hsa-mir-215-5p	0.0299	0.0330	0.0154	0.0003	0.4903	0.0104
hsa-mir-9-5p	0.0142	0.0237	0.0149	0.0129	0.7840	0.6712
hsa-mir-126-5p	0.0469	0.0321	0.0143	0.0071	0.3619	0.3048
hsa-mir-27b-5p	0.0135	0.0152	0.0138	0.0221	0.9576	1.5223
hsa-mir-374a-5p	0.0242	0.0169	0.0132	0.0131	0.6428	0.8714
hsa-mir-625-3p	0.0242	0.0262	0.0132	0.0008	0.5241	0.0431
hsa-mir-151a-5p	0.0185	0.0181	0.0129	0.0072	0.7027	0.4625
hsa-mir-185-5p	0.0220	0.0220	0.0127	0.0556	0.5749	3.2112
hsa-mir-141-5p	0.0231	0.0254	0.0118	0.0081	0.4881	0.4374
hsa-mir-106b-3p	0.0128	0.0194	0.0116	0.0086	0.7166	0.5522
hsa-mir-320a-3p	0.0262	0.0253	0.0105	0.0093	0.4083	0.5199
hsa-mir-339-3p	0.0227	0.0279	0.0099	0.0542	0.3910	2.8658
hsa-mir-15b-5p	0.0178	0.0237	0.0099	0.0245	0.4778	1.4620
hsa-mir-374b-5p	0.0178	0.0101	0.0094	0.0121	0.6700	1.2460
hsa-mir-671-5p	0.0355	0.0313	0.0094	0.0040	0.2799	0.1955
hsa-mir-331-3p	0.0135	0.0169	0.0077	0.0007	0.5064	0.0608
hsa-mir-18a-5p	0.0213	0.0194	0.0066	0.0049	0.3238	0.3784
hsa-let-7c-5p	0.1178	0.0934	0.0065	0.0962	0.0620	1.9246
hsa-mir-181a-3p	0.0142	0.0144	0.0061	0.0069	0.4233	0.6729
hsa-mir-342-3p	0.1507	0.1319	0.0061	0.0108	0.0428	0.1573
